# Feasibility and safety of robot-assisted thoracic surgery for lung lobectomy in patients with non-small cell lung cancer: a systematic review and meta-analysis

**DOI:** 10.1186/s12957-017-1168-6

**Published:** 2017-05-08

**Authors:** Shiyou Wei, Minghao Chen, Nan Chen, Lunxu Liu

**Affiliations:** 1 Department of Thoracic surgery, West China Hospital, Sichuan University, No. 37, Guoxue Alley, Chengdu, Sichuan 610041 China; 2 Center for Reproductive Medicine, Guangdong Women and Children Hospital, Guangzhou, 510010 China; 3 Western China Collaborative Innovation Center for Early Diagnosis and Multidisciplinary Therapy of Lung Cancer, Sichuan University, No. 37, Guoxue Alley, Chengdu, Sichuan 610041 China

**Keywords:** Robot-assisted thoracic surgery, Video-assisted thoracic surgery, Minimally invasive surgery, Lung lobectomy

## Abstract

**Background:**

The aim of this study is to evaluate the feasibility and safety of robot-assisted thoracic surgery (RATS) lobectomy versus video-assisted thoracic surgery (VATS) for lobectomy in patients with non-small cell lung cancer (NSCLC).

**Methods:**

An electronic search of six electronic databases was performed to identify relevant comparative studies. Meta-analysis was performed by pooling the results of reported incidence of overall morbidity, mortality, prolonged air leak, arrhythmia, and pneumonia between RATS and VATS lobectomy. Subgroup analysis was also conducted based on matched and unmatched cohort studies, if possible. Relative risks (RR) with their 95% confidence intervals (CI) were calculated by means of Revman version 5.3.

**Results:**

Twelve retrospective cohort studies were included, with a total of 60,959 patients. RATS lobectomy significantly reduced the mortality rate when compared with VATS lobectomy (RR = 0.54, 95% CI 0.38–0.77; *P* = 0.0006), but this was not consistent with the pooled result of six matched studies (RR = 0.12, 95% CI 0.01–1.07; *P* = 0.06). There was no significant difference in morbidity between the two approaches (RR = 0.97, 95% CI 0.85–1.12; *P* = 0.70).

**Conclusions:**

RATS lobectomy is a feasible and safe technique and can achieve an equivalent short-term surgical efficacy when compared with VATS, but its cost effectiveness also should be taken into consideration.

## Background

Lobectomy is considered to be the standard therapy for patients with non-small cell lung cancer (NSCLC) at an early stage, and a minimally invasive approach such as video-assisted thoracic surgery (VATS), rather than thoracotomy, has been recommended to this group of patients [1]. Since the initial VATS lobectomy described in the early 1990s [2, 3], growing evidence has suggested that VATS is an appropriate approach, which shows superior perioperative outcomes and improved long-term survival for selected patients with early stage NSCLC when compared with conventional thoracotomy [4, 5]. Despite such demonstrated advantages of VATS, some shortcomings such as steep learning curve, difficult hand-eye coordination, lack of instrument flexibility, and two-dimensional vision might still restrict the development of this technique [6, 7].

Robot-assisted thoracic surgery (RATS) is a relatively new technique for minimally invasive lung lobectomy. And the initial feasibility and safety of RATS lobectomy have been described by several publications in the past 10 years [8–11]. RATS lobectomy appears to present some advantages of VATS approach in terms of decreased blood loss, less impairment in pulmonary function, and short hospital length of stay when compared to conventional thoracotomy [12–14]. However, RATS lobectomy may be limited by its potential longer operative time and higher hospital costs. Ignoring these disadvantages, advocates still emphasize the benefits of RATS in regard to three-dimensional high-definition view, improved ergonomics less steep learning curve, and better maneuverability of instruments [15, 16]. Unfortunately, there is lack of evidence-based information on the feasibility and safety of RATS lobectomy in patients with NSCLC and whether RATS lobectomy can achieve equivalent short-term surgical efficacy when compared with VATS is also unknown. Therefore, we conducted this systematic review and meta-analysis of published studies in an attempt to assess the feasibility and safety of RATS lobectomy versus those with VATS.

## Materials and methods

### Search strategy

Electronic searches were performed in PubMed, Embase, Web of Science, Cochrane Central Register of Controlled Trials, Cochrane Database of Systematic Reviews, and ClinicalTrials.gov up to December 2016 without language restriction. We combined the terms “VATS OR video-assisted thoracic surgery OR thoracoscopic surgery” and “robotics OR robot OR robotic surgery OR computer-assisted surgery OR da Vinci” to search for eligible comparative studies. References of included studies were also manually searched to identify potentially relevant studies.

### Study selection

Preferred reporting items for systematic reviews and meta-analyses (PRISMA) flow chart was adapted to depict the study selection process [17]. After removing duplicates, two reviewers (SYW and MHC) independently reviewed the relevant studies by checking the titles, abstracts, and full-texts. Studies were eligible for inclusion in this meta-analysis if they were randomized or non-randomized controlled trials comparing RATS to VATS. We excluded studies which were relevant to RATS wedge resection or segmentectomy and those which did not contain a comparative group. In the case of duplicate publications with accumulating numbers of patients or increased lengths of follow-up, we only included the most recent or complete reports for our analysis.

### Data extraction

Two reviewers (SYW and MHC) independently examined the included studies and extracted data points pertaining to first author’s name, year of publication, study design, study period, surgical technique for RATS or VATS lobectomy, preoperative patient demographics (number of patients, geographic location, lobe distribution, and pathological stage), intraoperative parameters (operative time, blood loss, and conversion), and postoperative parameters (dissected lymph nodes station and number, hospital length of stay, prolonged air leak, arrhythmia, pneumonia, composite morbidity, perioperative mortality, and costs). The primary outcomes were perioperative mortality and morbidity, and the secondary outcomes were operative time, blood loss, hospital length of stay, prolonged air leak, arrhythmia, pneumonia, conversion, dissected lymph nodes (LNs) station and number, and costs. Discrepancies were resolved by group discussion and consensus with a senior investigator (LXL).

### Quality assessment

The risk of bias for each included observational study was assessed using the Newcastle-Ottawa Scale (NOS). The NOS includes three parts for cohort studies: selection (four scores assigned), comparability (two scores assigned), and outcome (three scores assigned). Studies with scores of 0 to 3, 4 to 6, and 7 to 9 were considered to be low, moderate, and high quality, respectively.

### Statistical analysis

Meta-analysis was performed by pooling the results of reported incidence of overall morbidity, mortality, prolonged air leak, arrhythmia, and pneumonia. Subgroup analysis was also conducted based on matched and unmatched cohort studies, if possible. Relative risks (RR) and their 95% confidence intervals (CI) were calculated for discontinuous data. Summary RRs were calculated by using fixed-effect models when heterogeneity among studies was considered to be statistically insignificant. Otherwise, random-effect models were used to combine the results. Heterogeneity among the studies was identified by conducting a standard Cochrane’s Q test with a significance level of *α* = 0.10. The *I* ^2^ statistic test was performed to further examine heterogeneity. *I* ^2^ ≥ 50% was considered to indicate substantial heterogeneity. Besides, visual inspection of the funnel plots was used to identify potential publication bias. All *P* values were two-tailed, and *P* < 0.05 was considered to be statistically significant. All analysis was conducted with Review Manager Version 5.3 (Cochrane Collaboration, Software Update, Oxford, United Kingdom, 2014).

## Results

### Literature search

**Fig. 1:**
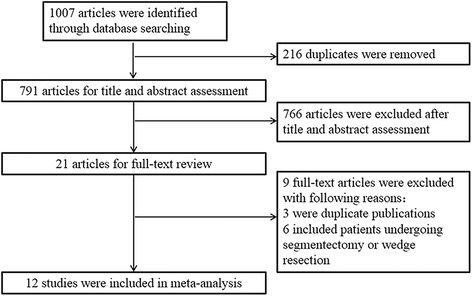
The PRISMA flow chart of selection for included studies

The initial search identified 1007 references. After duplicates were removed, 791 articles were retrieved for title and abstract assessment, and 21 articles were selected for full-text evaluation. Nine articles were excluded; of which, three articles were duplicate publications and six articles were relevant to RATS segmentectomy or wedge resection. Finally, a total of 12 retrospective cohort studies were included in this systematic review and meta-analysis [18–29]. The flow chart of selection for included studies is depicted in Fig. 1.

### Characteristics and risk of bias assessment

The included studies were published from 2010 to 2017. Of the 12 studies, seven studies [21, 23–26, 28, 29] were conducted in North America, two [22, 27] in Europe, two [18, 19] in Asia, and one [20] in Australia. Overall, 60,959 patients were identified for the analysis; of whom, 4727 patients underwent RATS and 56,232 patients received VATS lobectomy. The average age across various studies ranged from 26 to 88 years old. Of the 12 included studies, eight [18–22, 26–28] referred to the surgical technique of RATS, seven [18–21, 26–28] reported the arms of the da Vinci surgical system, and only five [18, 21, 26–28] provided information about the type of da Vinci surgical system.

The quality of the included studies assessed by the NOS was generally acceptable, with a mean NOS scores of 6.8 (standard deviation = 0.7). For most included studies, the methodological quality in terms of cohort selection and comparability was adequate. However, the follow-up periods were limited for most studies except for two studies [26, 28]. The characteristics and risk of bias assessment of the included studies were shown in Table 1

**Table 1:** Characteristics of included studies

Author and year	Location	Study design	Study period	No. of patients	Da Vinci robotic system	Surgical techniques	Surgeons	Lebo distribution (right/left)	Pathologic stage (I/II/III, IV)	NOS scores
Jang et al. (2011) [18]	Korea	RCS	RATS: 2009, VATS: 2008–2009	RATS (*n* = 40), VATS (*n* = 40)	da Vinci S system	RATS: four-arm RAL; VATS: four-port VATS	Same	RATS: 23/17, VATS: 26/14	RATS: 22/8/7, VATS: 31/3/6	6
Lee et al.(2012) [19]	Korea	RCS	RATS: 2008–2011, VATS: 2008–2011	RATS (*n* = 100), VATS (*n* = 100)	NA	RATS: four-arm RAL; VATS: three ports VATS	Same	RATS: NA, VATS: NA	RATS: 69/20/11; VATS: 80/12/8	7
Augustin et al.(2013) [20]	Australia	RCS	RATS: 2001–2008, VATS: 2009	RATS (*n* = 26), VATS (*n* = 26)	NA	RATS: three-arm RAL; VATS: three ports VATS	Different	RATS: 16/10, VATS: 12/14	RATS: 23/1/0; VATS: 18/2/5	7
Adams et al. (2014) [21]	USA	RCS	RATS: 2010–2012, VATS: 2009–2010	RATS (*n* = 120), VATS (*n* = 4612)	da Vinci Si system	RATS: four-arm CPRL; VATS: NA	NA	RATS: NA, VATS: NA	RATS: NA, VATS: NA	7
He et al. (2014) [22]	UK	RCS	RATS: 2012, VATS: 2012	RATS (*n* = 30), VATS (*n* = 34)	NA	RATS: RAL; VATS: NA	NA	RATS: NA, VATS: NA	RATS: NA, VATS: NA	6
Kent et al. (2014) [23]	USA	RCS	RATS: 2008–2010, VATS: 2008–2010	RATS (*n* = 340), VATS (*n* = 1020)	NA	RATS: NA; VATS: NA	Different	RATS: NA, VATS: NA	RATS: NA, VATS: NA	6
Paul et al. (2014) [24]	USA	RCS	RATS: 2008–2011, VATS: 2008–2011	RATS (*n* = 2498), VATS (*n* = 37,595)	NA	RATS: NA; VATS: NA	NA	RATS: NA, VATS: NA	RATS: NA, VATS: NA	6
Swanson et al. (2014) [25]	USA	RCS	RATS: 2009–2011, VATS: 2009–2011	RATS (*n* = 100), VATS (*n* = 100)	NA	RATS: NA; VATS: NA	NA	RATS: NA, VATS: NA	RATS: NA, VATS: NA	7
Lee et al. (2015) [26]	USA	RCS	RATS: 2012–2014, VATS: 2009–2014	RATS (*n* = 53), VATS (*n* = 158)	da Vinci S system	RATS: four-arm CPRL; VATS: two-port VATS	Different	RATS: 34/19, VATS: 99/59	RATS: NA, VATS: NA	8
Mahieu et al. (2016) [27]	France	RCS	RATS: 2012–2013, VATS: 2009–2010	RATS (*n* = 28), VATS (*n* = 28)	da Vinci S system	RATS: three-arm CPRL; VATS: three ports VATS	Same	RATS: 12/6, VATS: 15/8	RATS: 8/5/4, VATS: 11/5/8	7
Yang et al. (2017) [28]	USA	RCS	RATS: 2002–2012, VATS: 2002–2012	RATS (*n* = 172), VATS (*n* = 141)	da Vinci system	RATS: three-arm or four-arm; VATS: NA	Different	RATS: 110/62, VATS: 88/53	RATS: 133/29/10, VATS: 114/21/6	8
Louie et al. (2016) [29]	USA	RCS	RATS: 2009–2013, VATS: 2009–2013	RATS (*n* = 1220), VATS (*n* = 12,378)	NA	RATS: NA; VATS: NA	NA	RATS: 757/439, VATS: 7618/4648	RATS: NA, VATS: NA	7

*RCS* retrospective cohort study, *RATS* robot-assisted thoracic surgery, *VATS* video-assisted thoracic surgery, *RAL* robot-assisted lobectomy, *CPRL* completely portal robot lobectomy, *NA* not available

### Assessment of perioperative outcomes

**Fig. 2:**
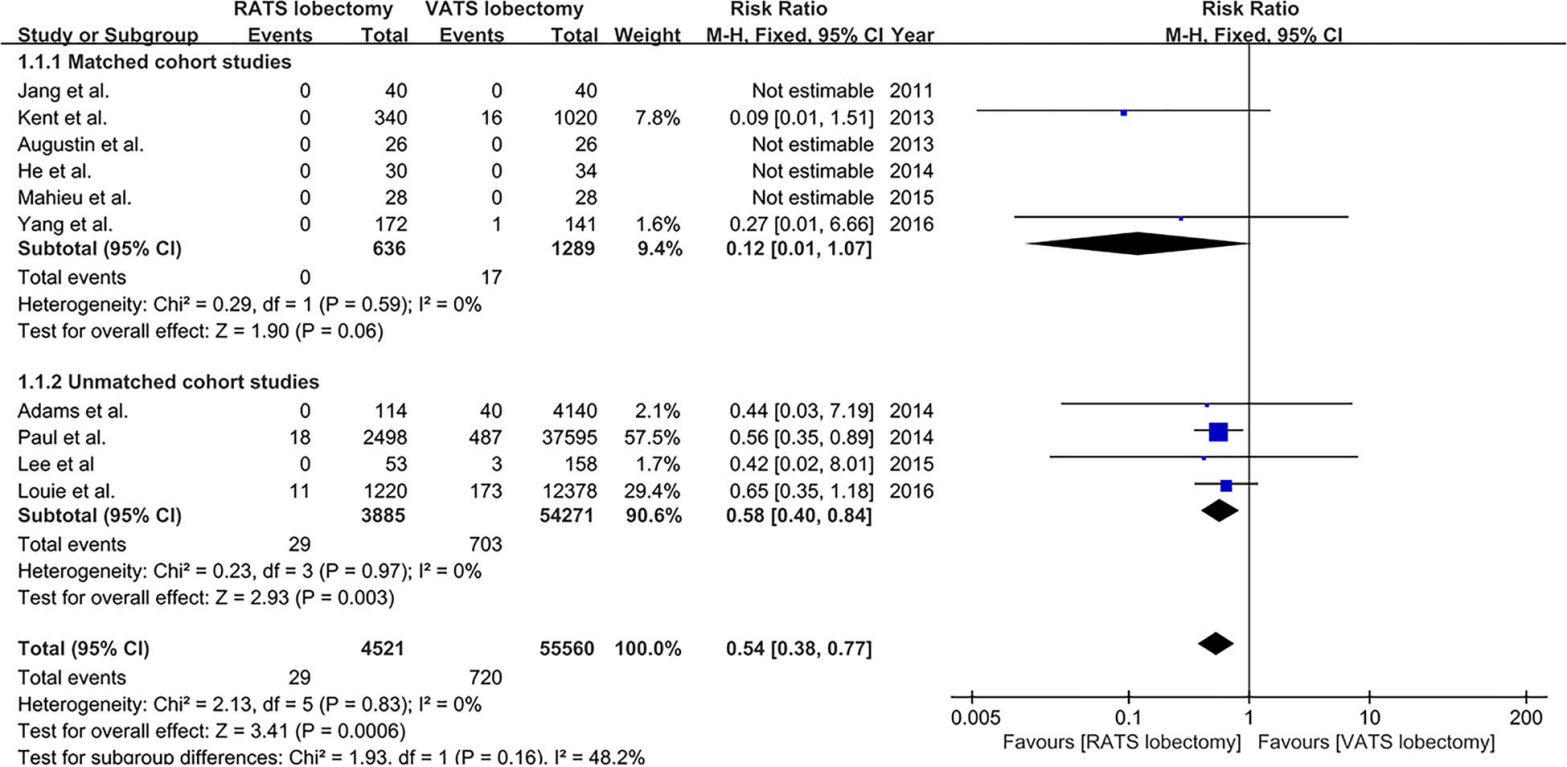
The forest plot and meta-analysis of mortality for patients undergoing RATS versus VATS lobectomy

A total of ten studies [18, 20–24, 26–29] that compared RATS to VATS lobectomy reported perioperative mortality outcome, including six matched studies [18, 20, 22, 23, 27, 28] and four unmatched studies [21, 24, 26, 29]. Mortality was 0.6% (29/4521) and 1.3% (720/55,560) for patients undergoing RATS and VATS, respectively. The pooled analysis of mortality demonstrated that when compared to VATS lobectomy, RATS showed a significantly lower mortality (RR = 0.54, 95% CI 0.38–0.77; *P* = 0.0006; fixed model), and this result was in line with the pooled result of three unmatched studies (RR = 0.58, 95% CI 0.40–0.84; *P* = 0.003), but was not consistent with the pooled result of six matched studies (RR = 0.12, 95% CI 0.01–1.07; *P* = 0.06) (Fig. 2). There was no statistical heterogeneity among the studies (*I* ^2^ = 0%, *P* = 0.83). Visual inspection of the funnel plots did not identify a potential publication bias.

**Fig. 3:**
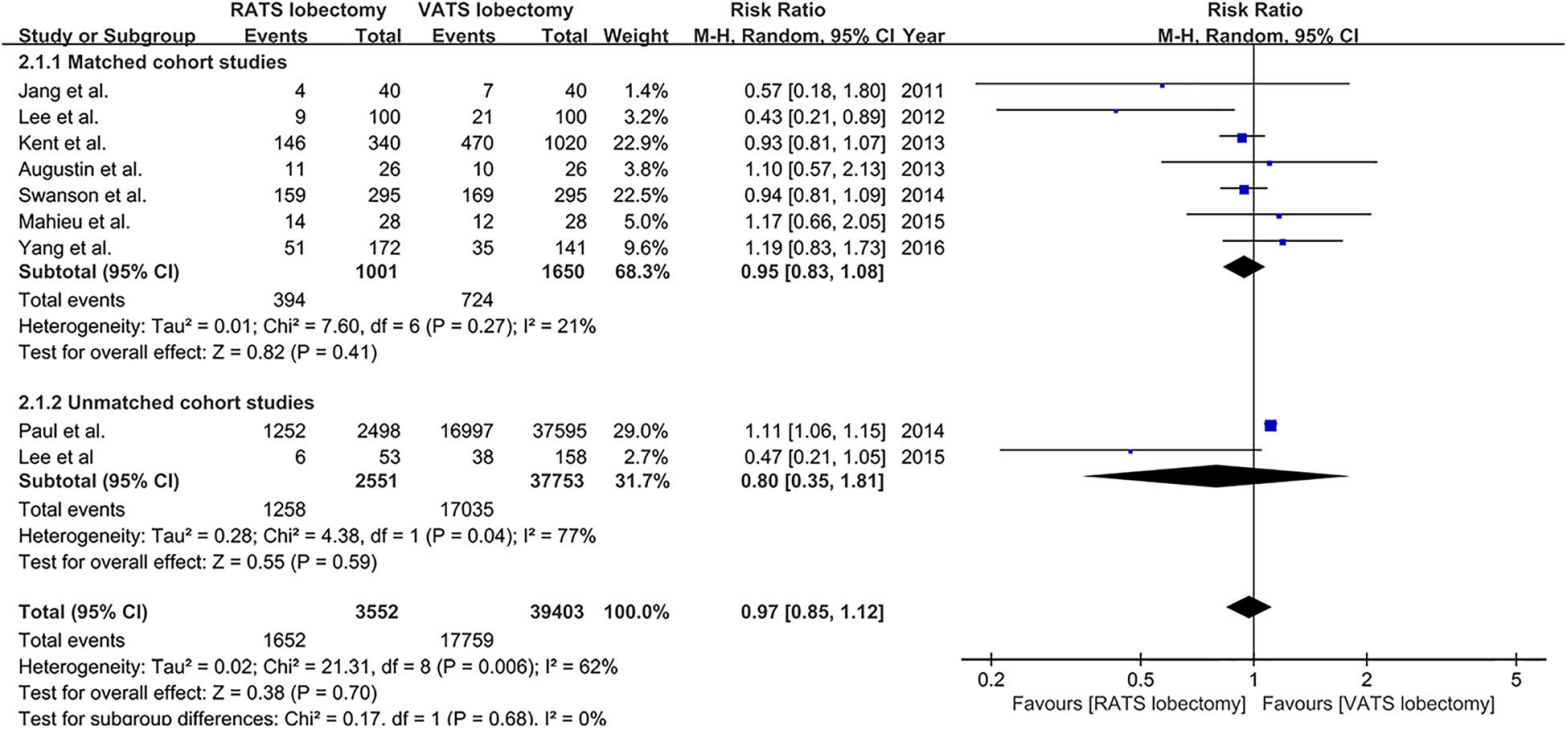
The forest plot and meta-analysis of composite morbidity for patients undergoing RATS versus VATS lobectomy

Composite morbidity was reported in nine studies [18–20, 23–28] (seven matched studies [18–20, 23, 25, 27, 28] and two unmatched studies [24, 26]). The overall morbidity rate was 46.5% (1652/3552) and 45.1% (17,759/39,403) in patients who underwent RATS and VATS lobectomy, respectively. The result of meta-analysis revealed that there was no statistically significant difference in composite morbidity between RATS and VATS lobectomy (RR = 0.97, 95% CI 0.85–1.12; *P* = 0.70; random model), and there was a significant heterogeneity among the eight studies (*I* ^2^ = 62%, *P* = 0.006) (Fig. 3). Publication bias was not evident from visual inspection of the funnel plots.

**Fig. 4:**
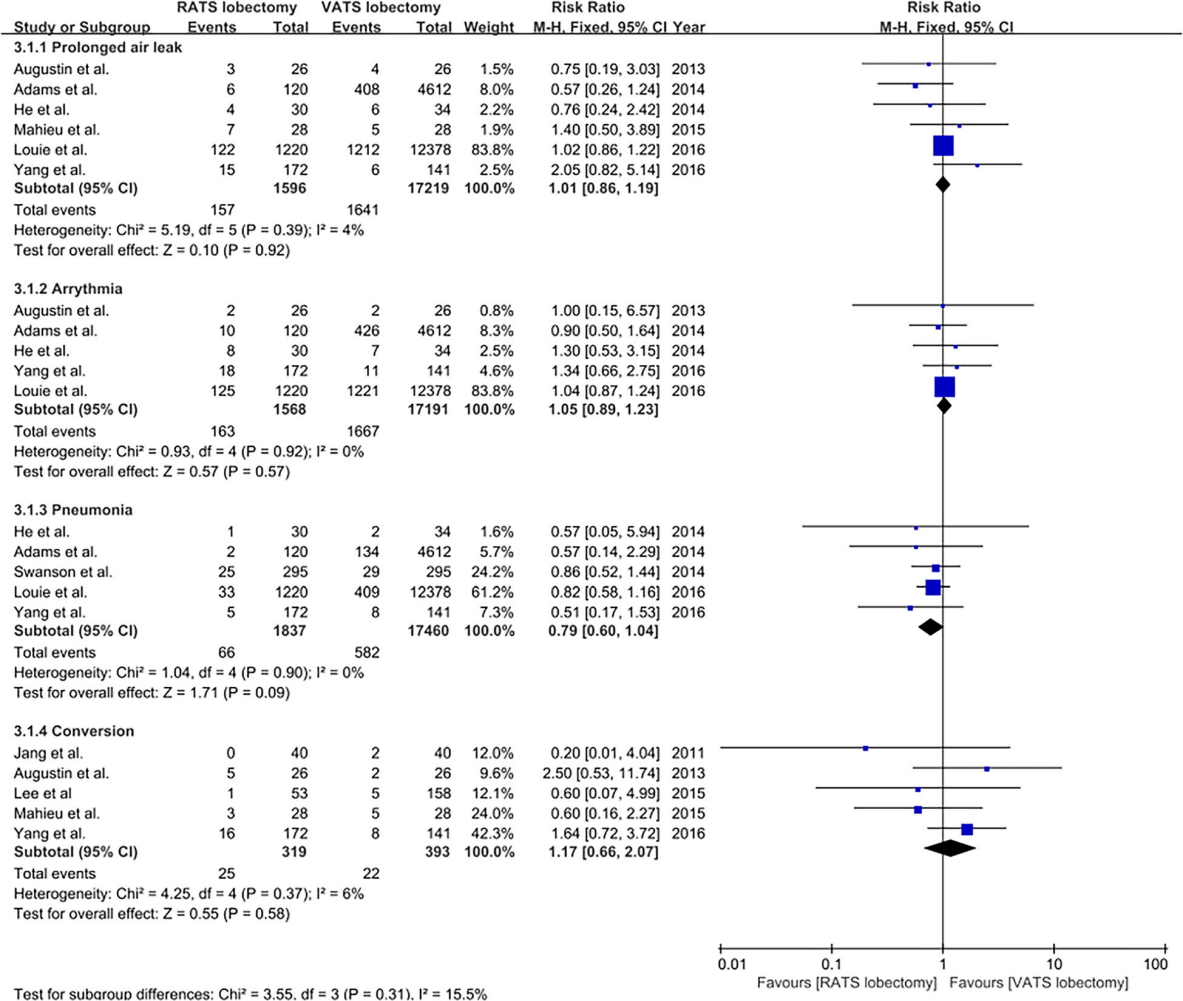
The forest plot and meta-analysis of subtype morbidity for patients undergoing RATS versus VATS lobectomy

Prolonged air leak was reported in six studies [20–22, 27–29], and the incidence of prolonged air leak was 9.8% (157/1596) and 9.5% (1641/17,219) for patients undergoing VATS and RATS, respectively. The incidence of arrhythmia that was reported in five studies [20–22, 28, 29] was 10.4% (163/1568) for RATS lobectomy and 9.7% (1667/17,191) for VATS lobectomy. Five studies [21, 22, 25, 28, 29] reported the data on the incidence of pneumonia, which was 3.6% (66/1837) and 3.3% (582/17,460) for patients undergoing RATS and VATS lobectomy, respectively. Five studies [18, 20, 26–28] provided the rate of conversion, and the incidence of conversion was 7.8% (25/319) and 5.6% (22/393) for RATS and VATS lobectomy, respectively. The meta-analysis on prolonged air leak, arrhythmia, pneumonia, and conversion all showed no significant differences between RATS and VATS lobectomy (prolonged air leak RR = 1.01, 95% CI 0.86–1.19, *P* = 0.92; arrhythmia RR = 1.05, 95% CI 0.89–1.23, *P* = 0.57; pneumonia RR = 0.79, 95% CI 0.60–1.04, *P* = 0.09; conversion RR = 1.17, 95% CI 0.66–2.07, *P* = 0.58; fixed model) (Fig. 4).

For the 12 studies [18–29] that compared RATS to VATS lobectomy, operative time was significantly longer in RATS group in six studies [18–21, 26, 29], shorter in one study [22], no difference in two studies [25, 27], and not reported in three studies [23, 24, 28]. No significant difference was found in blood loss between RATS and VATS lobectomy in two studies [18, 27]. Only one study [19] showed a significant shorter hospital length of stay when comparing RATS to VATS lobectomy, and ten studies [18, 20, 21, 23–29] did not observed a difference between the two approaches. The number of dissected lymph nodes station ranged from five to seven and the number of removed lymph nodes ranged from 14 to 24 for RATS lobectomy, which were comparable to VATS lobectomy. However, costs were significantly increased for RATS lobectomy in the included studies (Table 2).

**Table 2:** Perioperative outcomes of included studies

Study	Operative time (min)		Blood loss (ml)		Hospital length of stay (d)		Dissected LNs station		Dissected LNs number		Costs	
	RATS	VATS	RATS	VATS	RATS	VATS	RATS	VATS	RATS	VATS	RATS	VATS
Jang et al.	240	161*	219	245	6	7	7	8	22	26*	NA	NA
Lee et al. (2012)	209	157*	NA	NA	6.3*	8.9	7.3	6.0	24.8	23.6	NA	NA
Augustin et al.	215	183*	NA	NA	11	9	NA	NA	NA	NA	2507€	1736€
Adams et al.	241	179.8*	NA	NA	4.7	5.3	4.1	NA	9.4	NA	NA	NA
He et al.	145.50*	162.79	NA	NA	NA	NA	NA	NA	NA	NA	NA	NA
Kent et al.	NA	NA	NA	NA	5.9	5.7	NA	NA	NA	NA	NA	NA
Paul et al.	NA	NA	NA	NA	5	5	NA	NA	NA	NA	$22,582	$17,874*
Swanson et al.	269.4	253.8	NA	NA	6.07	5.83	NA	NA	NA	NA	$25,041	$20,477*
Lee et al. (2015)	161	123*	NA	NA	3	3	NA	NA	17*	11	NA	NA
Mahieu et al.	190	185	100	200	6	7	5	3	14	14	NA	NA
Yang et al.	NA	NA	NA	NA	4	4	5*	3	NA	NA	NA	NA
Louie et al.	186	173*	NA	NA	4	4	NA	NA	NA	NA	NA	NA

*P < 0.05

*RATS* robot-assisted thoracic surgery, *VATS* video-assisted thoracic surgery, *LNs* lymph nodes, *NA* not available

## Discussion

Since the first use of the da Vinci robotic surgical system for pulmonary lobectomy which was reported in 2002 [30], several studies [8, 9] have showed the feasibility and safety of this novel technique for lobectomy. A systematic review performed by Cao et al. [31] showed that perioperative mortality for patients who underwent pulmonary resection by RATS ranged from 1 to 3.8% and overall morbidity ranged from 10 to 39%. However, Cao et al. did not conduct a pooled analysis to assess the safety and efficacy of RATS lobectomy compared to those of VATS lobectomy. In another study [32], eight retrospective observational studies were eligible for meta-analysis and were evaluated for perioperative morbidity and mortality, but the meta-analysis included patients who underwent lobectomy, segmentectomy, and wedge resection.

The present systematic review and meta-analysis identified twelve retrospective cohort studies, including a total of 60,959 patients who underwent RATS lobectomy (*n* = 4727) and VATS lobectomy (*n* = 56,232). The meta-analysis revealed that RATS lobectomy significantly reduced the mortality rate when compared with VATS lobectomy (RR = 0.54, 95% CI 0.38–0.77; *P* = 0.0006), but this was not consistent with the pooled result of six matched studies (RR = 0.12, 95% CI 0.01–1.07; *P* = 0.06). This result could be explained in part by the highly selected patients at the beginning of this surgical technique; therefore, this result should be interpreted with caution. Moreover, the overall perioperative morbidity rate of RATS was similar to that of VATS lobectomy, and no statistically significant differences were observed in the incidence of postoperative prolonged air leak, arrhythmia, and pneumonia when comparing RATS to VATS lobectomy. Anyway, these outcomes suggest that RATS lobectomy is a safe and feasible surgical approach for patients with lung cancer and can achieve an equivalent short-term surgical efficacy compared with VATS lobectomy.

With respect to the operative results, most included studies reported a longer operative time for RATS compared to VATS lobectomy [18–21, 26, 29]. This can be explained by several potential factors. First, the knowledge and experience of RATS lobectomy for surgeons were inadequate at the beginning of the learning curve and most included studies just reported their initial attempts to RATS lobectomy. Second, prolonged operative time was reported to be caused by setting up the robotic system [18, 20]. Third, different surgery approaches might lead to different operative time. As reported in the study of Augustin et al. [20], the overall operative time was longer in the RATS group, but when comparing the anterior approach of RATS to VATS lobectomy, there was no significant difference on operative time. But it should be mentioned that the increased operative time in robotic surgery did not seem to have a negative impact on postoperative results, since there was no increase in short-term morbidity and mortality for patients. Besides, operative time for RATS approach has been shown to significantly shorten after the initial learning period. Therefore, with the increased knowledge and experience of RATS, operative time for RATS would be comparable to VATS.

In addition, in our present study, higher costs for lung lobectomy with the da Vinci surgical system was observed in most included studies. In a large case series, Park et al. [33] demonstrated that RATS lobectomy was on average $3981 more expensive than VATS lobectomy, but $3988 cheaper than open lobectomy. And the increased costs of RATS compared with VATS lobectomy occurred primarily in the first hospital day, which could be explained as the additional disposable costs of the robotic instruments and a higher percentage of additional procedural costs. Augustin et al. [20] also indicated that two thirds of the additional costs for RATS lobectomy were caused by disposables and the use of robotic instruments. However, in a retrospective analysis of 176 RATS lobectomies compared to 76 VATS lobectomies, Dylewski et al. [34] showed that direct costs was reduced by $560 per case in RATS group. And the majority of costs saving benefited from reduced length of hospital stay and lower overall nursing care costs. In addition, according to Deen et al. [35], shortening operative time, eradicating unnecessary laboratory work, reducing respiratory therapy, and minimizing stays in the intensive care unit would contribute to a decrease of hospital costs for patients who underwent RATS lobectomy. However, the costs associated with the overall substantial acquisition and maintenance for the robotic system was usually not included in analysis in most studies. In fact, a da Vinci robotic surgical system currently costs between $1 and $2.5 million in the United States [36], and is associated with annual maintenance costs of approximately $100,000 to $170,000 [33, 37]. Therefore, in the Japanese health care system, it is necessary to perform at least 300 robotic operations per year in each institution to avoid financial deficit with the current process of robotic surgical system management [38]. Since the effectiveness of RATS lobectomy is equivalent with increased costs when compared with that of VATS procedure, manufacturers of robotic surgical system would reduce supply costs by developing new generation robotic system to be more competitive.

There are several limitations existing in the present systematic review and meta-analysis. Firstly, it should be acknowledged that the data included in the present meta-analysis were extrapolated from 12 retrospective cohort studies. Although the heterogeneity was negligible among the included studies, selection bias of retrospective studies may lead to unbalanced selection of patients. Secondly, the characteristics of included patients and the surgical techniques were not clearly described in some included studies, which may lead a bias for the meta-analysis results. Thirdly, specific criteria for the definition of outcomes, such as prolonged air leak, conversion, and morbidity, were not clearly stated in most included studies. Fourthly, there are lacks of long-term follow-up outcomes for the comparison of RATS lobectomy with VATS lobectomy. Hence, future studies should emphasize the rigorous eligibility criteria, clear definition of outcomes, and long-term follow-up data.

## Conclusions

In conclusion, the current systematic and meta-analysis demonstrates that RATS lobectomy is a feasible and safe technique for selected patients and can achieve an equivalent short-term surgical efficacy when compared with VATS procedure. However, longer operative time and cost effectiveness of RATS should be taken into consideration, and long-term oncological efficacy of the RATS approach remains to be seen.

## Abbreviations

CI: Confidence interval
CPRL: Completely portal robot lobectomy
LNs: Lymph nodes
NA: Not available
NOS: Newcastle-Ottawa Scale
NSCLC: Non-small cell lung cancer
RAL: Robot-assisted lobectomy
RATS: Robot-assisted thoracic surgery
RCS: Retrospective cohort study
RR: Relative risks
VATS: Video-assisted thoracic surgery.
